# Cytotoxicity of Different Composite Resins on Human Gingival Fibroblast Cell Lines

**DOI:** 10.3390/biomimetics6020026

**Published:** 2021-04-20

**Authors:** Riccardo Beltrami, Marco Colombo, Keren Rizzo, Alessio Di Cristofaro, Claudio Poggio, Giampiero Pietrocola

**Affiliations:** 1 Department of Clinical-Surgical, Diagnostic and Pediatric Sciences—Section of Dentistry, University of Pavia, 27100 Pavia, Italy; marco.colombo@unipv.it (M.C.); keren.rizzo01@universitadipavia.it (K.R.); 2 Unit of Biochemestry, Departement of Molecular Medicine, University of Pavia, 27100 Pavia, Italy; alessiodc-94@hotmail.it (A.D.C.); giampiero.pietrocola@unipv.it (G.P.)

**Keywords:** cytotoxicity, gingival fibroblast, MTT test, composite resins

## Abstract

The aim of the present study was to evaluate and compare the cytotoxic effects of eight composite resins on immortalized human gingival fibroblasts. Composite resins were eluted in cell culture medium for 48 or 72 h at 37 °C. Immortalized human gingival fibroblast-1 (HGF-1) cell lines were seeded in 96-well (1 × 10^4^) plates and incubated for 24 h at 37 °C with the obtained extraction medium. The percentage of viable cells in each well (MTT test) was calculated relative to control cells, which were set to 100%. Data observed were not normally distributed, and nonparametric statistical methods were used for statistical analysis. The Wilcoxon test was used for intragroup comparison, and the Kruskal–Wallis test was used for intergroup multiple comparisons. Significance value was set as *p* < 0.05. All materials tested showed cytotoxic effects on gingival fibroblasts, recordable as noncytotoxic, mildly cytotoxic or severely cytotoxic, depending on the percentage of cell viability. The Wilcoxon test for intragroup comparison showed that the percentage of viable cells decreased significantly for extracts, for all composite resins tested. The composite resins contained monomers that displayed cytotoxic properties. BisGMA, TEGDMA and UDMA had inhibitory effects and induced apoptotic proteins in pulp fibroblast. Composite resins that contained lower percentages of unbound free monomers—and that released less ions—possessed superior biocompatibility in vitro.

## 1. Introduction

Composite resins are used extensively as restorative materials in conservative dentistry because of their ideal mechanical properties and desirable aesthetics. However, their use requires specific focus on the safety of the components used [1,2,3]. Over the last twenty years, resin composites have been developed in order to reduce cytotoxicity and polymerization shrinkage and improve aesthetics [1,2]. The biocompatibility of composites is an issue that requires particular attention to be paid to the chemistry of the biopolymers from which they are made [3]. Innovative resin composites are composed of a polymerizable organic resin matrix and a particulate ceramic reinforcing filler. These two main components are connected by a silane coupling agent [4]. The attention on biocompatibility is focused mainly on the polymerizable organic resin matrix. Among the components of the composite resins, the resin matrix is the only unstable one—primarily because of the unbound monomers that could be released. One study [3] stated that 15–50% of the methacrylic groups in the organic matrix remained as free monomers during the polymerization phase [5]. The amount of organic resin matrix used has been reduced over time. Composite resins have now evolved from hybrid polymers to organically modified ceramic materials (ormocers). Recent studies [6,7] on ormocers reported unacceptable clinical performances over long-term behavior (as compared to conventional composites) [7]. Recently, manufacturers introduced nano-hybrid ormocers in order to maintain high standards in the physicochemical properties of the materials [1,2,8]. According to the composition data provided, resin matrix consists of methacrylate-functionalized polysiloxanes with added silicate oxide. Manufacturers have stated that this asset of composition frees less unbound monomers, thus resulting in a higher biocompatibility of composite material [9,10,11]. The present study aimed to improve knowledge about the biocompatibility of different nano-hybrid composite resins by testing their cytotoxicity on immortalized human gingival fibroblast-1 HGF-1 (ATCC CRL-2014) cell lines using cell viability assay.

## 2. Materials and Methods

Eight composites were selected for this study: Omnichroma (OC), Omnichroma Blocker (OCB), Admira Fusion x-tra (AFX), Enamel Plus HRi Bio Function Enamel (EPE), Enamel Plus HRi (EP), G-aenial (anterior) (GA), G-aenial Flo X (GFX), Enamel Plus HRi Bio Function Bio Dentine (EPD).

The specifications of the materials are listed in Table 1.

### 2.1. Cell Culture

Immortalized human gingival fibroblast-1 HGF-1 (ATCC CRL-2014) cell lines were obtained from the American Type Culture Collection and cultured in high-glucose Dulbecco’s modified Eagle’s medium (DMEM; Sigma-Aldrich, St. Louis, MO, USA) supplemented with 4 mM L-glutamine (Sigma-Aldrich), 1% penicillin, streptomycin (Sigma-Aldrich), and 10% (*v/v*) heat-inactivated fetal bovine serum (FBS; Sigma-Aldrich). Cells were incubated at 37 °C in 5% CO_2_ atmosphere, fed every 48 h and routinely subcultured every 5 days with a split ratio of 1:3 using trypsin-EDTA (0.05%; Sigma-Aldrich) for 3 min at 37 °C.

### 2.2. Sample Preparation

Composite disc samples with a diameter of 7 mm and a height of 2 mm were prepared according to ISO 10993-12:2012 standards using customized molds, consistent with the manufacturers’ instructions [12,13]. While condensation of the unpolymerized composite was achieved on a glass plate, a mylar matrix strip was applied on the surface to limit oxygen inhibition. Excess material was removed with a sterile scalpel. Polymerization was accomplished using an LED light source (Celalux 3, High-Power LED curing-light; VOCO, Cuxaven, Germany) at an average 720 mW/cm^2^ for 40 s, applied to bottom and top surfaces of the disc. The composite disc samples (*n* = 3) were then UV sterilized prior to cytotoxicity testing. Excess material was removed with a sterile scalpel. To prevent contamination, specimens were exposed to UV light for 24 h after manipulation. Each composite was immersed in extraction medium immediately after setting.

### 2.3. Preparation of the Extract

The extraction was made by eluting the composites in cell culture medium (see cell culture paragraph) using a surface area-to-volume ratio of approximately 1.25 cm^2^/mL between the surface of the samples and the volume of medium [12]. The extraction vials were incubated at 37 °C for 48 h or 72 h. The specimens were then discarded, and the elute extracts were filtered by 0.22-μm-pore-sized membranes (Millipore; Billerica, MA, USA). Undiluted extracts were used for the cytotoxicity tests.

### 2.4. Cytotoxicity Test

Cells (1 × 10^4^) were seeded in each well of a 96-well plate and incubated for 24 h at 37 °C. Cultures were then exposed to 100 μL of the extract medium. Fresh cell medium was used as control. After 24 h, cell viability was determined using MTT assay. The MTT solution—(3-{4,5-dimethylthiazol-2-yl}-2,5-diphenyl tetrazolium bromide) (Sigma-Aldrich) in RPMI-1640 without phenol red (Sigma-Aldrich) (5 mg/mL)—was added to each well of the culture plate to reach a final concentration of 0.5 mg/mL, and the cells were incubated for 4 h at 37 °C. Then, the supernatant was removed and the resulting formazan was dissolved by adding 100 μL DMSO (Sigma-Aldrich) to each well. The optical density of formazan dye was read at 545 nm against 620 nm as background by an Elisa reader (Bio-Rad, Hercules, California, USA). The percentage of viable cells in each well was calculated relative to control cells set to 100%. Cytotoxicity responses were rated as severe (30%), moderate (30–60%), mild (60–90%) or noncytotoxic (>90%) [4,5,10].

### 2.5. Statistical Analysis

The control group had a cytotoxicity volume of 0% and viability volume of 100%. Data were analyzed with R (The R Foundation for Statistical Computing). The resulting data (Table 2) were expressed as percentages of cell viability rates, where the control group correlated to about 100%. Descriptive statistic values—median, minimum, maximum, mean and standard deviation—were calculated. Data observed were not normally distributed and nonparametric statistical methods were used for statistical analysis. The Wilcoxon test was used for intragroup comparison, and the Kruskal–Wallis test was used for intergroup multiple comparisons. Significance value was set as *p* < 0.05.

## 3. Results

**Figure 1:**
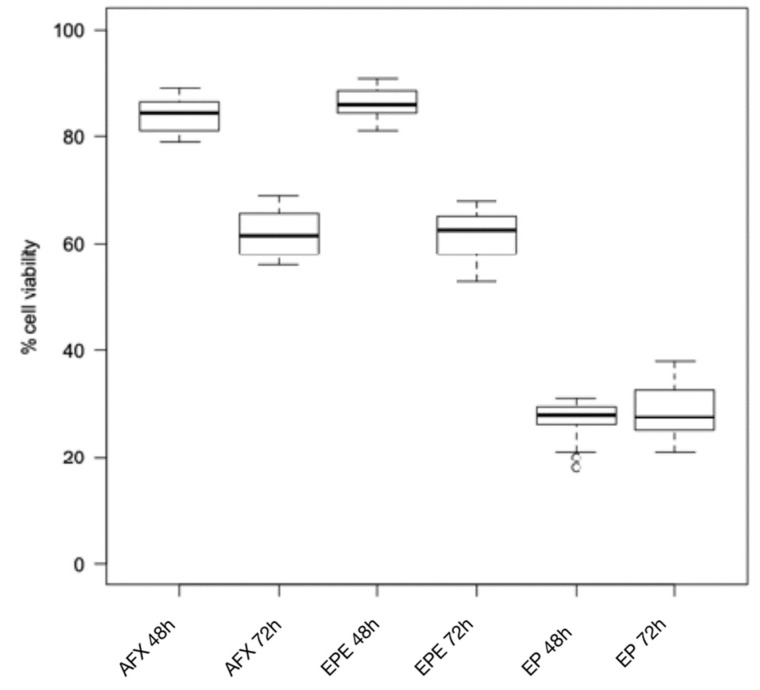
Cytotoxicity of composite resins on human gingival fibroblast cell lines after 24 h and 48 h. AFX: Admira Fusion x-tra; EPE: Enamel Plus HRi Bio Function Enamel; EP: Enamel Plus HRi.

**Figure 2:**
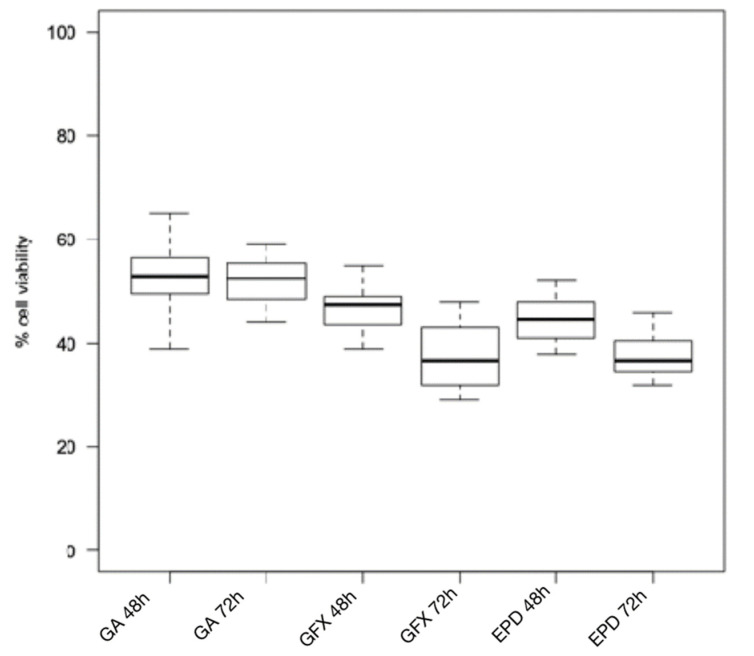
Cytotoxicity of composite resins on human gingival fibroblast cell lines after 24 h and 48 h. GA: G-ænial (Anterior); GFX: G-ænial Flo X; EPD: Enamel Plus HRi Bio Function Bio Dentine.

**Figure 3:**
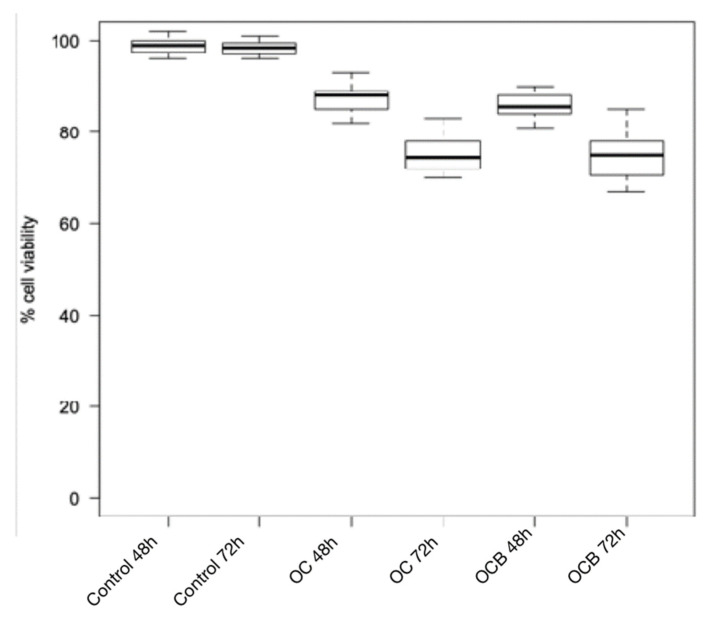
Cytotoxicity of composite resins on human gingival fibroblast cell lines after 24 h and 48 h. OC: Omnichroma; OCB: Omnichroma Blocker.

The Wilcoxon test for intragroup comparison showed that, after 72 h of incubation, the cell viability rates were significantly lower than after 48 h of incubation for all the composite resins tested except for Enamel Plus HRi and G-aenial (Figure 1 and Figure 2). However, Enamel Plus HRi and G-aenial showed, respectively, severe and moderate toxicity after 48 h. The cell viability rates maintained equally after 72 h. After 48 h, Omnichroma, Omnichroma Blocker, Admira Fusion x-tra, and Enamel Plus HRi Bio Function Enamel showed the lowest grade of cytotoxicity (cell viability > 80%) and no significant differences were recorder for intergroup multiple comparisons with Kruskal Wallis test (Figure 3). After 72 h Omnichroma and Omnichroma Blocker showed a comparable mild cytotoxicity (Kruskal Wallis *p* > 0.05), with a significant decrease in cell viability rates as compared to data after 48 h (Wilcoxon *p* < 0.05). Admira Fusion x-tra and Enamel Plus HRi Bio Function Enamel showed a significant reduction to moderate cytotoxicity and cell viability rates were comparable with Kruskal Wallis test after 72 h (*p* > 0.05). G-aenial Flo X and Enamel Plus HRi Bio Function Bio Dentine showed similar results after 48 h and 72 h (Kruskal–Wallis *p* > 0.05). Both the composite resins showed a lower cell viability rate after 72 h as compared to 48 h immersion (Wilcoxon *p* < 0.05).

## 4. Discussion

Several authors have demonstrated that tooth restorations produce an inflammatory response in soft tissues adjacent to the restorative material [14,15,16]. The healthy oral tissue is covered by squamous epithelia of keratinocytes, and the connecting tissue underneath is formed by gingival fibroblasts. Over time, aesthetic demands from patients drew attention to issues regarding the inflammatory conditions of periodontal tissues in contact with dental restorations. The importance of biocompatibility of the dental material is crucial in cases of deep subgingival caries and abrasive defects of the root after gingival recessions. The aesthetic limits of resin-based materials were even correlated to unsatisfactory long-term durability of restorations due to secondary caries and microleakage. Composite resins have been reinforced with higher amounts of filler and different-sized filler particles. Manufacturers developed nano-hybrid composites with reduced free unbound monomers and higher amounts of silicate oxide, thus improving the physical properties of the composite resins. However, biocompatibility remains the key factor in the success or failure of restorations in many clinical cases. The importance of testing the ultimate composite resins and their components in vitro cannot be overstated; such tests allow the clinician to choose the best-suited material in every clinical case, avoiding inflammation of periodontal tissue and rapid decay of tooth restorations.

The principle of the direct contact in vitro test used to assess the biocompatibility of restorative materials is the direct contact between cell lines and the material, similarly to what happens in clinical situations. The effects of the monomers released after polymerization on the surrounding tissues is surface-dependent. In a validly reproduced direct contact test, sample surface area and culture medium volume will have an impact on the results [17]. In one study, the sample surface area of each disc was about 120 mm^2^ wider than the mean surface area in clinical fillings. Monitoring the molecular processes and cellular viability over a chosen time made it possible to determine the cytotoxicity of composite resins [12]. Several authors have stated that the direct contact test provided more sensitive results in the determination of material toxicity [18,19].

The present study aimed to compare (in vitro) the impact of nano-hybrid composites and composite resins on human fibroblasts. The limited results found in the literature did not clarify how composition influenced cytotoxic effects. Nano-hybrid ormocers release fewer monomers and show less cytotoxicity [20,21,22]. Yang et al. [20] found the lowest cytotoxicity on human fibroblasts in nano-hybrid ormocer groups. In our study, we found that Omnichroma and Omnichroma Blocker had a significantly lower cytotoxicity after 72 h of direct contact between the material and human fibroblasts. Continuous release of free monomers was registered for Admira Fusion x-tra and Enamel Plus HRi Bio Function Enamel. Cell viability rates decreased significantly between the first registration of the cell viability rate (after 48 h) and the second registration (after 72 h). An incomplete conversion from monomers to polymers could have been the cause of the decrease of cell viability rate observed. The degree of conversion from monomers to polymers depends on curing time [23,24]. Therefore, we assumed that the polymerization time (40 s) that we applied led to an even higher degree of conversion for ormocer composite materials and, consequently, to decreased cellular toxicity compared to Enamel Plus HRi, G-aenial (anterior), G-aenial Flo X, and Enamel Plus HRi Bio Function Bio Dentine, all of which exhibited a lower degree of conversion [25]. Additionally, the composite materials studied differed significantly in filler degree (Table 1). A high filler degree in a resin-based dental composite positively influences its physicomechanical properties [26]. Moreover, it minimizes the organic matrix, which improves the material’s biocompatibility [25]. The absence of classic resin monomers in Omnichroma, Omnichroma Blocker, Admira Fusion x-tra, and Enamel Plus HRi Bio Function Enamel apparently resulted in lower cytotoxicity levels and better biocompatibility compared to resin-based dental restorative materials. This may be of great importance for clinical use. A continuous release of monomers could be registered in further studies with longer experimental periods. Even if human cell viability and growth seem to be affected in the short term, a longer experimental period could help to investigate the chronic effects of unbound monomers on human cell lines [27]. The moderate–severe cytotoxicity expressed at the contact between resin-based composites and human gingival fibroblasts could increase if the duration of exposure were to increase in relation to the cumulative effect caused by continuous monomer release from the materials [27]. The contact between composite resins and human gingival fibroblasts in subgingival areas should be further investigated. However, this in vitro study—based on direct contact between restorative materials and human gingival fibroblasts—seemed to clarify the mechanisms of inflammation of periodontal tissues.

## 5. Conclusions

In this study, using MTT assay, we demonstrated that composite resins used for tooth restorations have different biocompatibility standards that depend mainly on composition and percentage of unbound monomers. Ceramic fillers—recently included in some restorative materials—could improve biocompatibility because less monomer is required to obtain a performant dental material. Clinicians should consider the inflammation that restorative materials could cause to surrounding periodontal tissue and consequently choose products that promote less cytotoxicity.

## Figures and Tables

**Table 1:** Composite resins tested in this study.

Material	Manufacturer	Code	Composition	Filler Content	Lot Number
Omnichroma	Tokuyama Dental Corporation Tokyo, Japan	OC	Matrix: UDMA/TEGDMA monomers Filler: spherical SiO_2_-ZrO_2_	79% (*w/w*)	003M2
Omnichroma Blocker	Tokuyama Dental Corporation Tokyo, Japan	OCB	Matrix: Bis-GMA, triethylene glycol dimethacrylate Filler: spherical SiO_2_-ZrO_2_	82% (*w/w*)	002
Admira Fusion x-tra	Voco, Cuxhaven, Germany	AFX	Matrix: ORMOCER^®^ Filler: glass ceramics, silica nanoparticles, pigments	84% (*w/w*)	1750435
Enamel Plus HRi Bio Function Enamel	Micerium S.p.A., Avegno, Italy	EPE	Matrix: urethane dimethacrylate, tricyclodecane dimethanol dimethacrylate, no comonomers and no Bis-GMA Filler: glass filler, high dispersion silicon dioxide, fluorine	74% (*w/w*)	2018006379
Enamel Plus HRi	Micerium S.p.A., Avegno, Italy	EP	Matrix: diurethan dimethacrylate, BisGMA, 1,4-butandioldimethacrylate Filler: surface-treated nano zirconium oxide particles, glass	77% (*w/w*)	2017008768
G-ænial (Anterior)	GC Corporation, Tokyo, Japan	GA	Matrix: UDMA, dimethacrylate co-monomers, no bis-GMa Filler: silica, strontium, lanthanoid fluoride	76% (*w/w*)	190530A
G-ænial Flo X	GC Corporation, Tokyo, Japan	GFX	Matrix: UDMA, Bis-MPEPP), TEGDMA Filler: silicon dioxide, strontium glass	71% (*w/w*)	190521A
Enamel Plus HRi Bio Function Bio Dentine	Micerium S.p.A., Avegno, Italy	EPD	Matrix: urethane dimethacrylate, tricyclodecane dimethanol dimethacrylate, no comonomers and no Bis-GMA Filler: glass filler, high dispersion silicon dioxide, fluorine	74 % (*w/w*)	2018006379

**Table 2:** Statistical comparisons using Wilcoxon Test for each material after 48 h and 72 h. Significance was set at 0.05.

Material	48 h		72 h		
	Median (Max–Min)	Mean (SD)	Median (Max–Min)	Mean (SD)	*p*
Control	99 (102–96)	98.8 (1.61)	98.5 (101–96)	98.4 (1.50)	0.448
Omnichroma	88 (93–82)	87.6 (3.14)	74.5 (83–70)	75.35 (3.67)	0.000
Omnichroma Blocker	85.5 (90–81)	85.8 (2.80)	75 (85–67)	74.65 (4.66)	0.000
Admira Fusion x-tra	84.5 (89–79)	84.3 (3.19)	61.5 (69–56)	61.85 (4.22)	0.000
Enamel Plus HRi Bio Function Enamel	86 (91–81)	86.2 (2.71)	62.5 (68–53)	61.5 (4.5)	0.000
Enamel Plus HRi	28 (31–18)	27,1 (3.6)	27.5 (38–21)	28.55 (4.85)	0.794
G-aenial (Anterior)	53 (65–39)	53.05 (5.52)	52.5 (59–44)	51.75 (4.63)	0.400
G-aenial Flo X	47.5 (55–39)	46.9 (4.14)	36.5 (48–29)	37.15 (5.19)	0.000
Enamel Plus HRi Bio Function Bio Dentine	44.5(52–38)	44.35 (4.2)	36.5 (46–32)	37.55 (4.1)	0.000

## Data Availability

The data presented in this study are available on request from the corresponding author.
